# Supplementary material for: Altitudinal variation in rhizosphere microbial communities of the endangered plant Lilium tsingtauense and the environmental factors driving this variation
Source: Microbiol Spectr. 2024 Oct 9;12(11):e00966-24. doi: 10.1128/spectrum.00966-24 (PMC11536999; doi:10.1128/spectrum.00966-24)
Supplement: Supplemental figures — Fig. S1 to S6. [file spectrum.00966-24-s0001.docx]

**Supplementary Materials**

**Altitudinal variation in rhizosphere microbial communities of the endangered plant *Lilium tsingtauense* and the environmental factors driving this variation**

Boda Liu, Jinming Yang, Wanpei Lu, Hai Wang, Xuebin Song, Shaobo Yu, Qingchao Liu, Yingkun Sun*, Xinqiang Jiang*

*Authors for correspondence:

Xinqiang Jiang, E-mail: jiangxinqiang8@163.com, Tel: +86 13553079586

Yingkun Sun, Email: 200001033@qau.edu.cn, Tel: +86 15653217278

**Supplementary Figures**

**
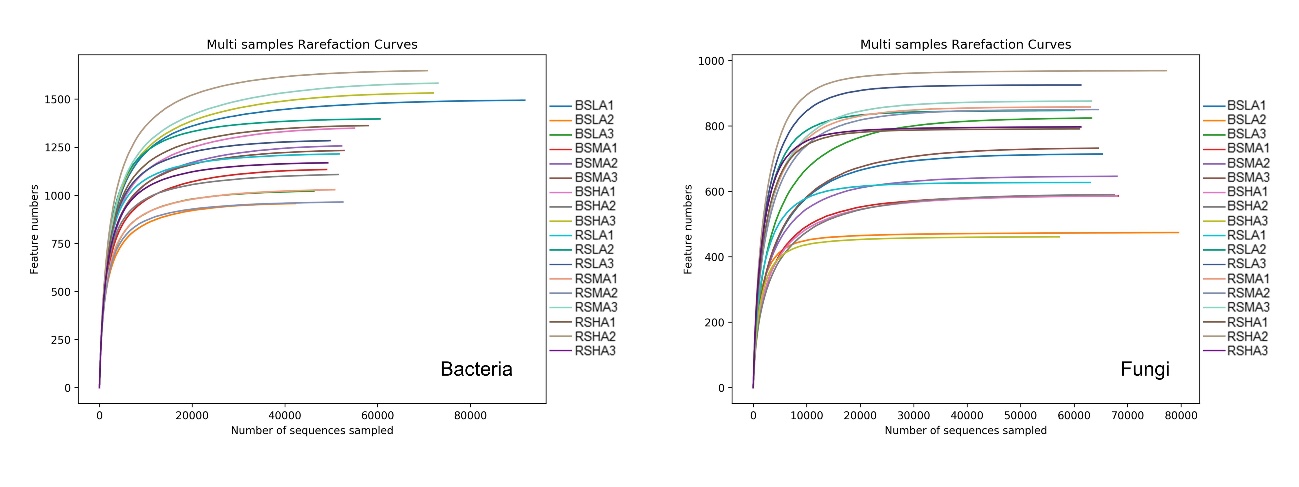
**

**FIG S1** Rarefaction Curve of rhizosphere (RS) and bulk soil (BS) microbial community (Bacteria and Fungi) of *L. tsingtauense*. All tests (n=3) analyses were repeated three times biologically. LA, low altitude; MA, middle altitude; HA, high altitude; RS, rhizosphere soil; BS, bulk soil.


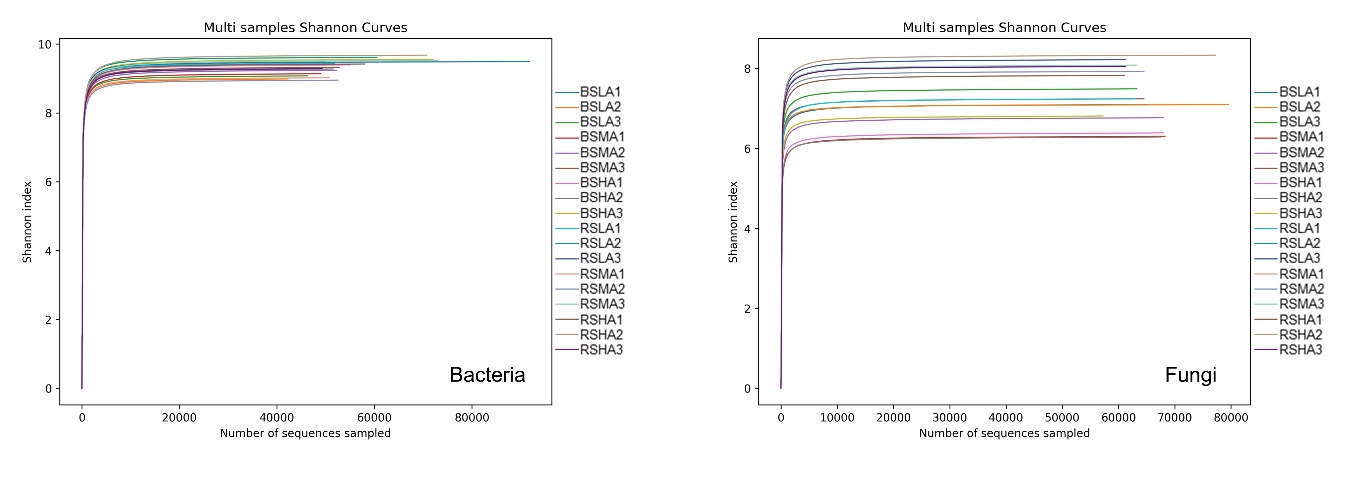


**FIG S2** Shannon Curve of rhizosphere (RS) and bulk soil (BS) microbial community (Bacteria and Fungi) of *L. tsingtauense*. LA, low altitude; MA, middle altitude; HA, high altitude; RS, rhizosphere soil; BS, bulk soil. All tests (n=3) analyses were repeated three times biologically.

**
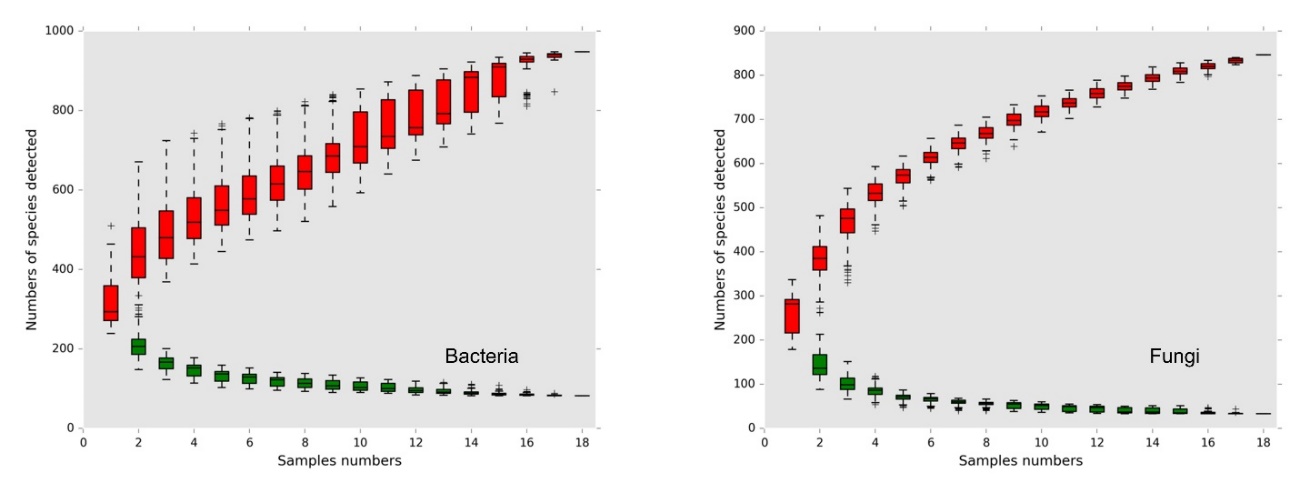
**

**FIG S3** Cumulative curve of relative abundance of species of rhizosphere (RS) and bulk soil (BS) microbial community (Bacteria and Fungi) of *L. tsingtauense*. A single red box reflects the total number of species contained in the sample, and the total red box constitutes the cumulative curve. A single green box reflects the number of common species in the sample, and the total green box composition common volume curve.


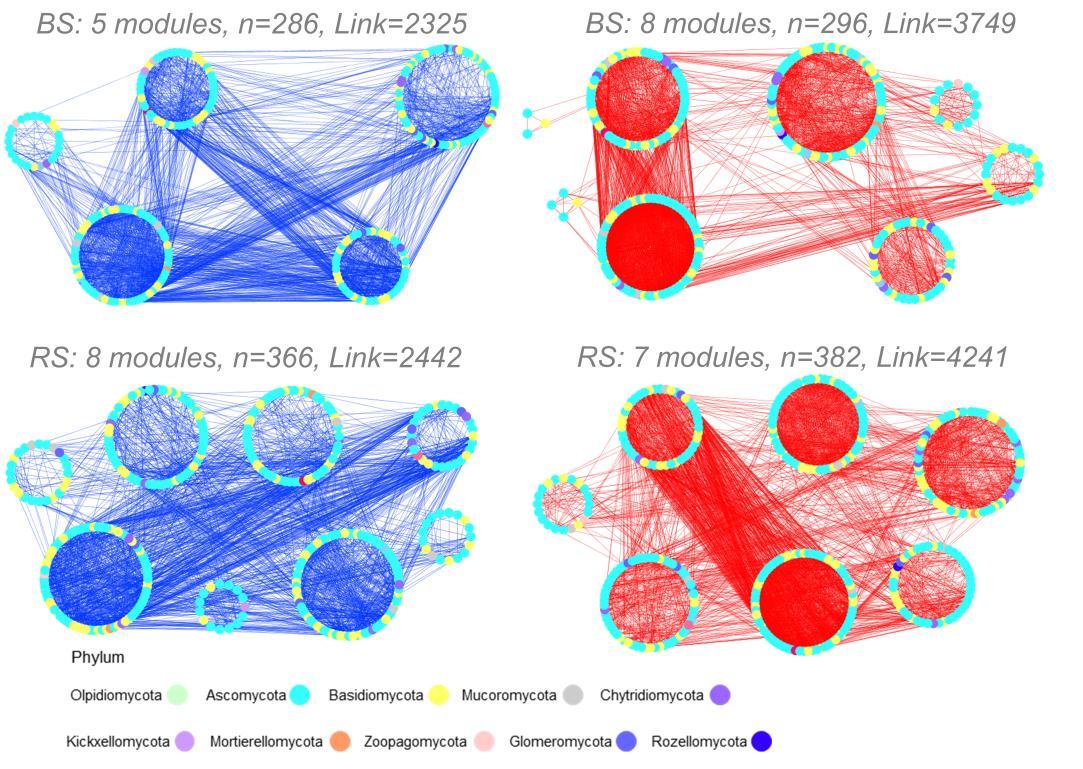


**FIG S4** Visualized networks of bacterial co-occurrence in rhizosphere (RS) and bulk soils (BS). The blue line represents a negative correlation, the red represents a positive correlation, and the co-occurrence network shows ASVs with abundance more than 0.01%.


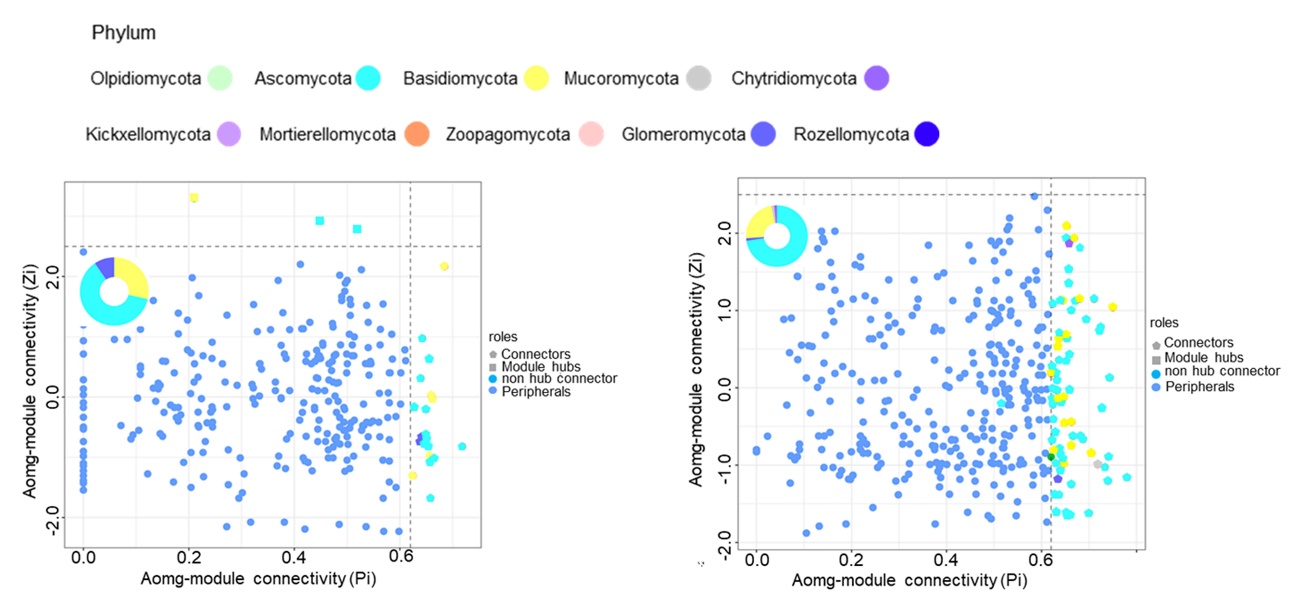


**FIG S5** Zi-Pi plot shows the distribution of ASVs based on their topological roles with bulk soil (BS) and rhizosphere (RS). All the nodes with Zi ≤ 2.5 or Pi ≥ 0.62 were determined as the keystone species, that is, nodes in the area of connectors, module hubs, and network hubs played a crucial role in the co-occurrence networks.


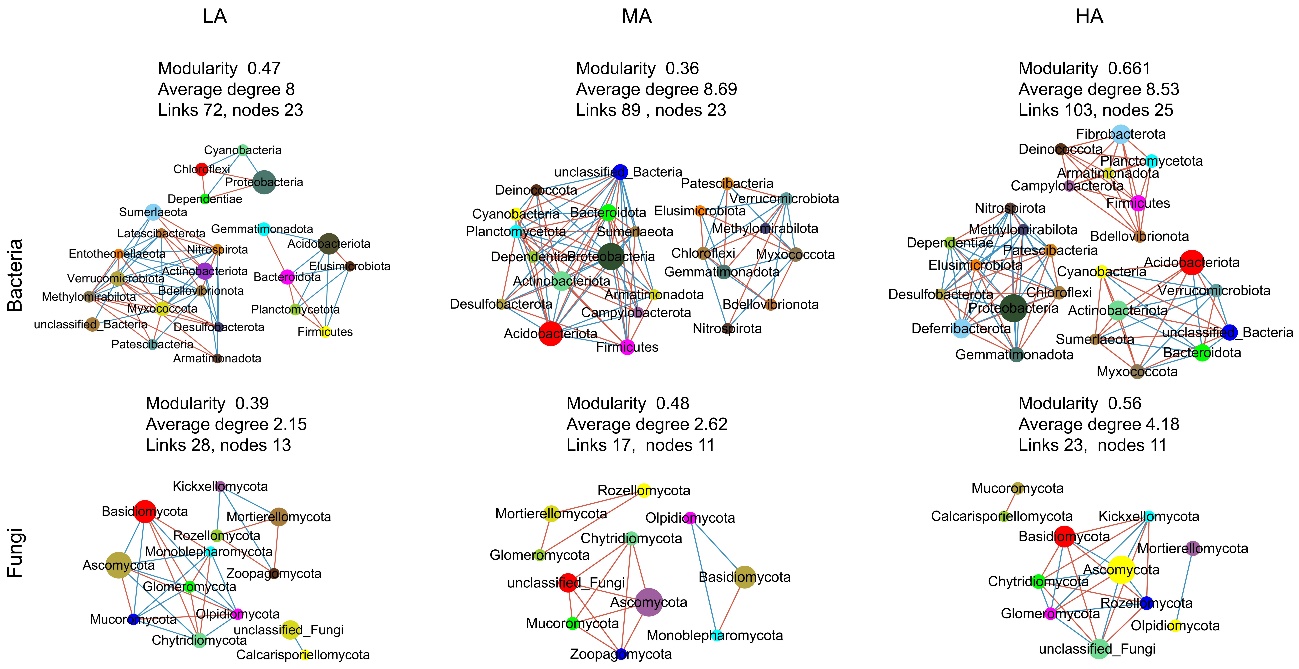


**FIG S6** Analysis of co-occurrence network (spearman, *P* < 0.05, r > 0.6) of bacteria and fungi in rhizosphere soil at three altitudes (LA, MA, HA). The size of nodes indicates the relative abundance of bacterial and fungal taxa. The red line shows a positive correlation; the blue line shows a negative correlation.
